# Hypovitaminosis D in migrant children in Switzerland: a retrospective study

**DOI:** 10.1007/s00431-021-04143-7

**Published:** 2021-06-15

**Authors:** Olivia Fahrni, Alexandra Wilhelm-Bals, Klara M. Posfay-Barbe, Noémie Wagner

**Affiliations:** 1 Faculty of Medicine, University of Geneva, Geneva, Switzerland; 2 Pediatric Nephrology Unit, Geneva University Hospitals and Faculty of Medicine, Geneva, Switzerland; 3 Pediatric Infectious Diseases Unit, Geneva University Hospitals and Faculty of Medicine, Geneva, Switzerland

**Keywords:** Vitamin D, Hypovitaminosis D, Supplementation, Children, Migrant, Refugee

## Abstract

Cholecalciferol (vitamin D_3_) is essentially known for its role in the phosphocalcic metabolism and its associated pathologies, such as rickets. In Switzerland, 35 to 50% of children are vitamin D deficient. Due to skin colour, poor nutrition, living conditions and cultural practices, migrant population is particularly at risk. Our aim is to attest the prevalence of hypovitaminosis D in children arriving in Switzerland. We retrospectively assessed 528 children’s vitamin D status and parathyroid hormone, phosphate and calcium levels between 2015 and 2018 by electrochemiluminescence and spectrophotometry. Cholecalciferol was considered insufficient under 50 nmol/L and severely deficient below 25 nmol/L. Seventy-three percent of children showed hypovitaminosis D and 28% had a severe deficiency. Highest prevalence of deficiency was found in children from Eastern Mediterranean (80%) and African regions (75%). Severe deficiency was more prevalent in the South East Asian (39%) and Eastern Mediterranean regions (33%) and more frequent in females. Deficiency was more frequent and more severe in winter. Hypovitaminosis D increased with age. Two children presented with all three biological manifestations associated to severe hypovitaminosis D (hyperparathyroidism, hypocalcaemia and hypophosphatemia).

***Conclusion***: A majority of migrant children presented with hypovitaminosis D. They should be supplemented to prevent complications. A strategy could be to supplement all children at arrival and during wintertime without regular vitamin D level checks.

**Table:** 

What is Known: *Hypovitaminosis D is frequent in children and can lead to bone-related complications.* *Migrant children are particularly at risk of deficiency.*	
What is New: *Three-quarters of migrant children evaluated at our migrant clinic in Geneva’s children hospital are deficient in vitamin D, one third severely.* *A strategy to correct the deficiency would be to supplement all migrant children at arrival and in winter.*

What is Known:

*Hypovitaminosis D is frequent in children and can lead to bone-related complications.*

*Migrant children are particularly at risk of deficiency.*

What is New:

*Three-quarters of migrant children evaluated at our migrant clinic in Geneva’s children hospital are deficient in vitamin D, one third severely.*

*A strategy to correct the deficiency would be to supplement all migrant children at arrival and in winter.*

## Introduction

Vitamin D_3_ (cholecalciferol) is classically known for its role in the calcium and phosphate metabolism. It is absorbed by ingestion (20%) or synthetized by the skin during sun exposure (80%) [1, 2]. In the setting of hypovitaminosis D, calcium and phosphate absorption will drop. Parathyroid hormone (PTH) will increase to normalize the calcium levels, resulting in normal to low serum calcium and phosphate and high PTH [3]. Severe deficiency can induce rickets or osteomalacia due to abnormal bone mineralization [2–4]. Vitamin D also seems involved in cancer, autoimmune and cardiovascular diseases prevention [3, 5–8] and, in children, hypovitaminosis D has additionally been associated to respiratory infections, asthma and eczema [1, 3, 8, 9].

Symptoms of hypovitaminosis D are variable, ranging from classical musculoskeletal to more subtle symptoms, such as irritability or tiredness, especially in teenagers. The more severe the deficit, the more symptomatic children will be [1].

In Switzerland, 35 to 50% of native children are deficient in vitamin D [10, 11]. Most of the vitamin D needed for health (80–90%) is produced by the skin as a result of ultraviolet radiation from the sun. In late autumn, winter and early spring, cloudy cover and weaker sun does not allow the skin to produce enough vitamin D. In summer, because of the increased use of solar cream to prevent skin cancer, skin exposure might also be insufficient. Moreover, fortified food is rare in Switzerland [1, 9, 10].

Due to nutritional deficiencies, cultural practices (type of clothing, time spent indoors, etc.) and darker skin phenotype (which, in turn, decreases cutaneous synthesis), migrant children are even more at risk [1, 12–15].

It is widely admitted that children older than one year old and teenagers should have a daily vitamin D intake of 600 IU (400 IU in infants younger than one) through sun, diet and, if necessary, supplementation [9, 16]. In case of severe or symptomatic deficiency, higher doses may be needed [1].

Specific supplementation recommendations vary between countries, notably due to differences in milk and food fortification in vitamin D. Swiss guidelines recommend a daily prophylaxis up to three years old and a daily intake of 600 IU for older children and teenagers, achieved through sun exposure, food or supplementation. In winter, as ultraviolet radiation is decreased in Switzerland, supplementation is recommended [10, 17]. Screening for vitamin D deficiency is also suggested in at-risk individuals, including dark-skinned people [1, 10]. Long-term compliance with daily supplementation seems however difficult to obtain [18]. Swiss paediatricians have the responsibility to individually assess if their patients can reach the recommended daily intake without supplementation according to their lifestyle and look for deficiency symptoms, in particular in case of risk factors.

In our institution, all migrant children are routinely screened for vitamin D deficiency at the first visit since 2015 and supplemented when necessary.

This study’s aim is to determine the prevalence of vitamin D deficiency in migrant children at arrival in Geneva, according to origin, age, gender and season.

## Materials and methods

### Study population

All migrant children arriving in Geneva are evaluated at a migrant clinic in Geneva’s Children Hospital. Since 2015, they benefit from a formal work-up during their first consultation, amongst which vitamin D, calcium, phosphate and PTH are measured. We retrospectively analysed the medical records of 1246 children between 2015 and 2018 to assess serum 25-hydroxyvitamin D (25(OH)D), calcium, phosphate and PTH.

Inclusion criteria were children aged 0–16 years old and first consultation between January 2015 and December 2018.

Exclusion criteria were born in Switzerland; prior stay in Switzerland; no 25(OH)D results and 25(OH)D measured later than 6 months after first consultation.

We also collected information about demographics, country of origin and season of blood exam. Four age categories (age at first consultation) were defined: < 3 years old, 3 to < 5 years old, 5 to < 10 years old and ≥ 10 years old.

Geographic zones followed the World Health Organization zones, except for Somalia, which was included in the African Region.

### Laboratory tests

Total vitamin D (25(OH)D_3_ and 25(OH)D_2_) and PTH were measured by chemiluminescent microparticle immunoassay and calcium and phosphate by spectrophotometry, using Roche Diagnostics kits (Switzerland) in our routine clinical laboratory. Accuracy and reliability of this immunoassay for vitamin D were assessed with a standardization program and in external quality assessment scheme.

Various cutoffs to stratify vitamin D levels exist. Following recent consensuses, we defined deficiency as a 25(OH)D concentration of ≤ 50 nmol/L (20 ng/ml) and severe deficiency as < 25 nmol/L (10 ng/ml) [16, 19]. These thresholds are based on bone outcomes with increased PTH below 50 nmol/L and risk of rickets/osteomalacia below 25 nmol/L [1]. Serum calcium was considered normal between 2.2 and 2.52 nmol/L and PTH between 1.1 and 6.8 pmol/L. Phosphate thresholds were adapted to age according to the CALIPER program [20, 21].

### Data collection and management

We retrospectively collected data in a protected and anonymous database. Patients with no 25(OH)D values were excluded from the study. Children with missing data for PTH, calcium and phosphate were excluded from the corresponding analysis. The ethics committee waived the need to collect individual patients’ consent. This study was approved by the institutional ethics committee on January 24, 2019 (study number: ID 2016-01278).

### Statistical analysis

We applied standard descriptive statistics. Groups were compared with χ^2^ tests. Linear regression was used to study the relationship between age and 25(OH)D levels. P values < 0.05 were considered statistically significant. Data were analysed with SPSS statistical software (version 25.0; SPSS Inc., Chicago, IL).

## Results

**Fig. 1:**
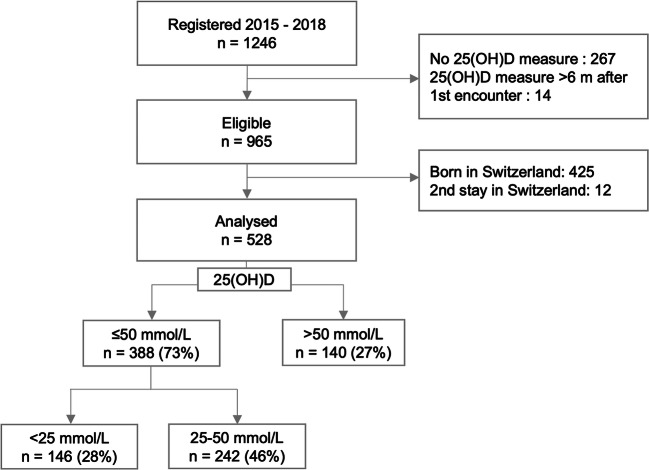
Flowchart showing excluded patients and vitamin D status of included children (25(OH)D levels < 25 nmol/L, 25–50 nmol/L, > 50 nmol/L)

Between 2015 and 2018, 1246 children had a first visit at Geneva’s migrant consultation. Five hundred twenty-eight were included (Fig. 1). Three hundred eighty-eight (73%) were deficient in vitamin D and 146 (28%) presented a severe deficiency.

Males represented 302 children (57%) and 226 were females (43%). Vitamin D deficiency was more frequent in females (77%) than males (71%), but no significant difference was found except for African girls (p = 0.03). Severe deficiency was significantly higher in females (34%) than males (23%) (p = 0.008), in particular if older than 10 years old (53% versus 31% respectively) (p = 0.01).

On average, the first consultation occurred 4.7 months after arrival in Switzerland (95%-CI = 3.94–5.46). 25(OH)D levels were examined on average 1.4 months after first encounter (95%-CI = 1.29–1.51).

**Fig. 2:**
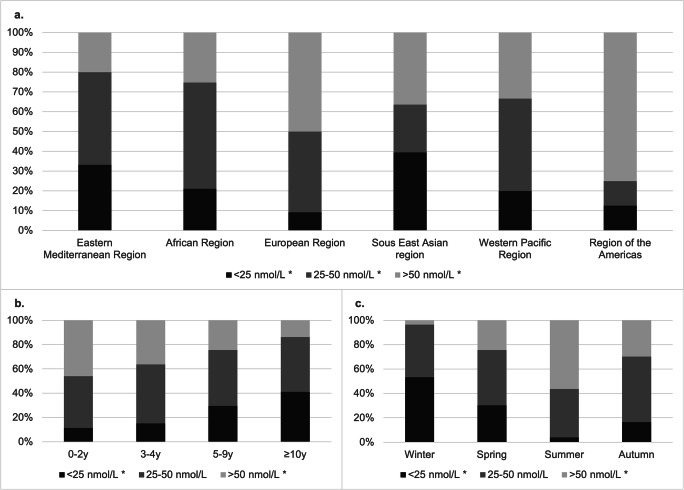
25(OH)D levels by geographic zones (**a**), age categories (**b**) and season of analysis (**c**). Graph (**a**) shows a significant difference in sufficiency (25(OH)D > 50 nmol/L ; p < 0.001), moderate deficiency (25(OH)D 25–50 nmol/L; p = 0.02) and severe deficiency (25(OH)D < 25 nmol/L; p = 0.001) between geographic zones. Graph (**b**) shows a significant increase in severe deficiency (p < 0.001) and concordant decrease in sufficiency (p < 0.001) with increasing age. Graph (**c**) shows a significant difference in severe deficiency (p < 0.001) prevailing in winter and sufficiency (p < 0.001) prevailing in summer. *p < 0.05

The most represented geographic zones were the Eastern Mediterranean (n = 295), African (n = 123) and European regions (n = 54). The highest prevalence of deficiency was found in the Eastern Mediterranean (80%) and African regions (75%). Severe deficiency was mostly found in the South East Asian (39%) and Eastern Mediterranean regions (33%). Children from the region of the Americas had predominantly within normal values (75%). Half of the European children were deficient (Table 1, Fig. 2a).

**Table 1:** Vitamin D status by origin

Origin (N)	25(OH)D ≤ 50 nmol/L (%)	25(OH)D < 25 nmol/L (%)	25(OH)D 25–50 nmol/L (%)	25(OH)D > 50 nmol/L (%)
**Eastern Mediterranean Region (295)**	**236 (80)**	**98 (33)**	**138 (47)**	**59 (20)**
Syria (133)	*98 (74)*	*26 (20)*	*72 (54)*	*35 (26)*
Afghanistan (67)	*57 (85)*	*31(46)*	*26 (39)*	*10 (15)*
Iraq (66)	*57 (86)*	*31 (47)*	*26 (39)*	*9 (14)*
Palestine (15)	*13 (87)*	*4 (27)*	*9 (60)*	*2 (13)*
Others^a^ (14)	*11 (79)*	*6 (43)*	*5 (36)*	*3 (21)*
**African Region (123)**	**92 (75)**	**26 (21)**	**66 (54)**	**31 (25)**
Eritrea (92)	*73 (79)*	*20 (22)*	*53 (58)*	*19 (21)*
Others^b^ (31)	*19 (61)*	*6 (19)*	*13 (42)*	*12 (39)*
**European Region (54)**	**27 (50)**	**5 (9)**	**22 (41)**	**27 (50)**
Georgia (20)	*9 (45)*	*2 (10)*	*7 (35)*	*11 (55)*
Others^c^ (34)	*18 (53)*	*3 (9)*	*15 (44)*	*16 (47)*
**South East Asian region (33)**	**21 (64)**	**13 (39)**	**8 (24)**	**12 (36)**
Sri-Lanka (32)	*21 (66)*	*13 (41)*	*8 (25)*	*11 (34)*
Others^d^ (1)	*0 (0)*	*0 (0)*	*0 (0)*	*1 (100)*
**Western Pacific Region (15)**	**10 (67)**	**3 (20)**	**7 (47)**	**5 (33)**
Mongolia (14)	*9 (64)*	*3 (21)*	*6 (43)*	*5 (36)*
Others^e^ (1)	*1 (100)*	*0 (0)*	*1 (100)*	*0 (0)*
**Region of the Americas (8)**	**2 (25)**	**1 (13)**	**1 (13)**	**6 (75)**
Others^f^ (8)	*2 (25)*	*1 (13)*	*1 (13)*	*6 (75)*
**Total (528)**	**388 (73)**	**146 (28)**	**242 (46)**	**140 (27)**

Regions are emphasised in bold. Subcategories of severe (25(OH)D < 25 nmol/L) and mild (25(OH)D 25-50 nmol/L) vitamin D deficiencies are emphasized in italic

^a^Iran, Egypt, Pakistan, Sudan, Yemen

^b^Algeria, Angola, Ethiopia, Somalia, Nigeria, Cameroon, Senegal, Burkina Faso, Ivory Coast, Guinea-Bissau, Uganda

^c^Turkey, Romania, Kosovo, Azerbaijan, Russia, Armenia, Macedonia, Serbia, Spain, Albania, Moldova, Portugal, Ukraine

^d^Bangladesh

^e^Philippines

^f^Bolivia, Salvador, Columbia, Honduras

Ninety (17%) children were born out of their country of origin. Amongst those, twenty-one were born out of their original geographic zone, with thirteen born in the European region.

The most represented countries of origin were Syria (n = 133), Eritrea (n = 92), Afghanistan (n = 67), Iraq (n = 66), Sri-Lanka (n = 32), Georgia (n = 20), Palestine (n = 15) and Mongolia (n = 14). Except for Georgia (45% of hypovitaminosis D), most children of the previously mentioned countries were deficient. Palestinian, Iraqi and Afghan children were the most deficient with more than 85% of insufficient values. Iraqi and Palestinian children showed the largest proportion of severe deficiency (47% and 46% respectively) (Table 1).

The predominant age categories were 5 to < 10 years old (n = 237) and ≥ 10 years old (n = 131). Children aged 3 to < 5 years old (n = 99) and < 3 years old (n = 66) were slightly less represented. Prevalence of vitamin D deficiency was > 50% in all age categories. Hypovitaminosis D increased with age (p < 0.001). More children in severe deficiency were also found with increasing age (p < 0.001) (Fig. 2b). There was no significant difference in the proportion of each gender within the age categories.

Seasonal vitamin D analysis was equally represented (103 measurements in summer, 152 in spring, 139 in winter and 134 in autumn). There was a difference in deficiency according to season (p < 0.001), with a predominance of insufficient values in winter (96%) and sufficient values in summer (56%). Severe deficiency was high in winter (53%) and almost inexistent in summer (4%) (Fig. 2c).

Ninety-one (20%) children had hyperparathyroidism with 90% concomitant hypovitaminosis D. Twenty-eight (6%) had hypocalcaemia with 89% concomitant vitamin D deficiency. Forty-two (9%) presented with hypophosphatemia with 86% concomitant deficient cholecalciferol. Two patients (0.5%) presented with simultaneous hyperparathyroidism, hypocalcaemia and hypophosphatemia, and both had concomitant severe vitamin D deficiency.

## Discussion

### Vitamin D levels

Three-quarters (73%) of migrant children are deficient in vitamin D at arrival in Switzerland, one-third (28%) of them severely. This is higher than the rate in Swiss children (35–50%) [10, 11]. It is consistent with other studies, although reference values for deficiency can vary between authors. For example, 72.3% of refugees in Canada were deficient (< 50 nmol/L) [22] and 77.4% in Italy (< 75 nmol/L) [23]. Supplementation is needed to prevent impact on bone health [1, 2, 4]

Besides the overall hypovitaminosis D, we demonstrated that the presence and severity of deficiency varies according to origin, age and gender. Even though hypovitaminosis D is classically associated with dark skin, a great majority of children with fairer complexion also presented with mild and severe deficiency. For example, Eastern Mediterranean children were predominantly deficient (80%), a third of them severely. In particular, Palestinian, Iraqi and Afghan children showed a large proportion of deficiency. This might be linked to cultural/religious practices associated with a more covering dress code. Another hypothesis may be related to the cause of migration (i.e. armed conflict might be associated with more time spent indoors).

As expected, African children were largely deficient (75%), although slightly less severely (21%). Similarly to Swiss children and adults, 50% of European children presented with hypovitaminosis D [10, 11]. This also mirrors the proportion of deficiency in European adults [24]. We found 75% of sufficient values in children from the Americas but these data are based on a very limited number of available patients (n = 8). Similar results for geographic distribution of hypovitaminosis D were found in a Canadian study [22]. Other studies in Norway and Italy reported comparable results, except for a lower prevalence of deficiency in the Western Pacific region (Norwegian study) and a higher prevalence of deficiency in America and Europe (Italian study) [23, 25]. However, the first study included adults and the reported countries were different. In the second study, vitamin D ranges were different and geographic zones not clearly defined.

Hypovitaminosis D significantly increased with age. Highest prevalence of deficiency (86%) and severe deficiency (41%) were found in children older than 10 years old. Other studies found concordant results in Italy [23] and Canada [22], with superior vitamin D status in younger patients.

Additionally, we found a significant difference in severe deficiency linked to gender, which was more frequent in females than males, especially if older than 10 years old. This result might be linked to cultural/religious dressing practices appearing with the first menstruations, limiting sun exposure. Gender differences in activities are also possible, with girls spending more time indoors than boys. A Canadian study also found that Middle East, Asian and African females were particularly at risk of hypovitaminosis D [22], whilst another study in Norway also showed a greater risk of hypovitaminosis D in females, especially if adolescent [25]. As pic bone mass occurs during puberty, this observation is crucial to ensure bone health and prevent fractures in late adulthood.

These results highlight the need for vitamin D prophylaxis for all migrant children and not only targeted to the young (< 3 years old) and dark-skinned children.

As expected due to the difference in sun exposure, vitamin D status largely varied between seasons, with a great majority of hypovitaminosis D in winter and a small majority of normal values in summer. Half of the children were severely deficient in winter. Although the specific impact of winter deficiency is not yet clearly understood, it seems to negatively affect bone health [26]. These results, consistent with current knowledge [9] and several studies [23, 27, 28], emphasize the importance of winter supplementation, in particular in the migrant population. Even though supplementation is recommended to Swiss children older than 3 years old if the daily intake of 600 IU is not met (e.g. in winter), it is often not prescribed by primary care physicians [10, 17]: this emphasizes the need for education to incorporate such guidelines in clinical practice.

Two children presented with simultaneous hyperparathyroidism, hypocalcaemia and hypophosphatemia. Both had concomitant severe vitamin D deficiency. Although rare in Europe, rickets is still present, especially in refugees and dark-skinned children and is mainly linked to vitamin D deficiency [29, 1].

Other biological manifestations related to vitamin D deficiency were found (hyperparathyroidism, hypocalcaemia and hypophosphatemia), all strongly correlated to hypovitaminosis D. These results were expected and emphasize the significant biological impact of decreased vitamin D levels.

Moreover, it underlines the importance of vitamin D dosage and supplementation in case of musculoskeletal symptoms or more subtle symptoms (irritability, tiredness, weakness), in particular in teenagers [1].

### Supplementation strategy

Our data suggest that vitamin D supplementation is highly needed for migrant children as 76% of the patients older than 3 years old presented with hypovitaminosis D, especially in winter. In the light of an uncostly vitamin D substitution compared to the vitamin D measurement and to avoid overdiagnosis [30], we would propose a systematic arrival supplementation (600 IU/day for three months) followed by winter substitution (two single doses of 100,000 IU/day) without vitamin D assessment. Both supplementation methods should not induce toxicity in children with normal vitamin status [31, 32], but, although less physiologic, we suggest the second approach for winter supplementation to increase compliance [1, 33].

## Strength and limitations

The first strength of our study is the relatively large number of participants with different regions of origin and both genders well covered. This grants a relatively good estimation of the vitamin D levels of the migrant population arriving in Geneva. Another strength is the simultaneous determination of calcium, phosphate and PTH, allowing a brief assessment of the biological impact of hypovitaminosis D. Finally, although gold standard is liquid chromatography-tandem mass spectrometry, our technique of vitamin D measurement by chemiluminescent immunoassay is a good method for assessing 25(OH)D, as subject to small intra- and interassay coefficient of variations [24].

However, this study is subject to various limitations. Although the number of patients in this study was relatively high, our population was heterogeneous. Some countries of origin and geographic zones were poorly represented. Furthermore, migrant population being subject to current refugee patterns, it might differ in other centres and change over time. Vitamin D levels might also vary according to type and duration of the refugee route taken or prior vitamin D substitution.

As our study was retrospective, we also lacked data about cultural/religious habits and outdoor time.

For these reasons, this study’s results and the supplementation strategy proposed should be confirmed in a larger and if possible multicentre trial and adapted locally.

## Conclusion

Three-quarters of migrant children arriving in Geneva are deficient in vitamin D and almost all of them have below-range levels in winter. Supplementation is needed to prevent hypovitaminosis D complications. Deficiency varies according to country of origin, geographic zone, age, gender and season of analysis but remains high in almost all categories. For this reason, we suggest to systematically prescribe vitamin D for migrant children older than three years old at arrival and every winter.

## Abbreviations

25(OH)D: 25-Hydroxyvitamin D
PTH: Parathyroid hormone
